# Low-Dose Vitamin D_3_ Supplementation Does Not Affect Natural Regulatory T Cell Population but Attenuates Seasonal Changes in T Cell-Produced IFN-γ: Results From the D-SIRe2 Randomized Controlled Trial

**DOI:** 10.3389/fimmu.2021.623087

**Published:** 2021-06-28

**Authors:** Wakunyambo Maboshe, Helen M. Macdonald, Heather Wassall, William D. Fraser, Jonathan C. Y. Tang, Shona Fielding, Robert N. Barker, Mark A. Vickers, Anthony Ormerod, Frank Thies

**Affiliations:** ^1^ School of Medicine, Medical Sciences and Nutrition, University of Aberdeen, Aberdeen, United Kingdom; ^2^ Norwich Medical School, University of East Anglia, Norwich, United Kingdom

**Keywords:** vitamin D, randomized control trial, T regulatory cells, interferon-gamma, seasonality, healthy subjects, immune markers

## Abstract

**Background:**

Seasonal variations have been reported for immune markers. However, the relative contributions of sunlight and vitamin D variability on such seasonal changes are unknown.

**Objective:**

This double-blind, randomized, placebo-controlled trial tested whether daily 400 IU vitamin D_3_ supplementation affected short-term (12 weeks) and long-term (43 weeks) natural regulatory T cell (nTreg) populations in healthy participants.

**Design:**

62 subjects were randomized equally to vitamin D versus placebo in March and assessed at baseline, April (4w), June (12w), September (25w) and January (43w). Circulating nTregs, *ex vivo* proliferation, IL-10 and IFN-γ productions were measured. Vitamin D metabolites and sunlight exposure were also assessed.

**Results:**

Mean serum 25-hydroxyvitamin D (25(OH)D) increased from 35.8(SD 3.0) to 65.3(2.6) nmol/L in April and remained above 75 nmol/L with vitamin D supplementation, whereas it increased from 36.4(3.2) to 49.8(3.5) nmol/L in June to fall back to 39.6(3.5) nmol/L in January with placebo. Immune markers varied similarly between groups according to the season, but independently of 25(OH)D. For nTregs, the mean (%CD3^+^CD4^+^CD127^lo^ cells (SEM)) nadir observed in March (2.9(0.1)%) peaked in September at 4.0(0.2)%. Mean T cell proliferation peaked in June (33156(1813) CPM) returning to the nadir in January (17965(978) CPM), while IL-10 peaked in June and reached its nadir in September (median (IQR) of 262(283) to (121(194) pg/ml, respectively). Vitamin D attenuated the seasonal increase in IFN-γ by ~28% with mean ng/ml (SEM) for placebo vs vitamin D, respectively, for April 12.5(1.4) vs 10.0(1.2) (p=0.02); June 13.9(1.3) vs 10.2(1.7) (p=0.02) and January 7.4(1.1) vs 6.0(1.1) (p=0.04).

**Conclusions:**

Daily low dose Vitamin D intake did not affect the nTregs population. There were seasonal variation in nTregs, proliferative response and cytokines, suggesting that environmental changes influence immune response, but the mechanism seems independent of vitamin D status. Vitamin D attenuated the seasonal change in T cell-produced IFN-γ, suggesting a decrease in effector response which could be associated with inflammation.

**Clinical Trial Registration:**

https://www.isrctn.com, identifier (ISRCTN 73114576).

## Introduction

Vitamin D deficiency has re-emerged as a global concern (1, 2). Due to low UVB availability, populations at high latitudes are predisposed to lower vitamin D levels (3). For bone health, UK government currently advises daily vitamin D intake of 10 μg (400 IU) throughout the year for the general population from 4 years old (SACN, 2016). It is unknown whether these are optimal for functions other than calcium homeostasis (4). Vitamin D deficiency may affect immune system functionality, leading to increased risk of severe infections, metabolic syndrome and autoimmunity (5, 6).

Research on vitamin D’s immunomodulatory effects primarily have focused on the *in vitro* effects of the active metabolite, 1,25(OH)_2_D, or its analogues (7, 8). Key targets for vitamin D include forkhead box P3 (FoxP3) transcription factor, the master regulator of regulatory T cells (Tregs) and interferon-gamma (IFN-γ) (9, 10). Tregs play a crucial role in immune homeostasis by regulating the magnitude of other immune cell responses (11) and maintaining self-tolerance (12, 13).

Few human intervention studies on the effects of vitamin D_3_ on Tregs and other immune markers have been conducted (14). Furthermore, these studies have either administered very high doses in healthy participants (15, 16) or used supplementation as a therapeutic agent in pre-existing disease (17, 18). Healthy participants supplemented with cholecalciferol (~5000 IU/day) over 12 weeks during wintertime showed a significant increase in Tregs (16), but it was not confirmed whether this was sustained throughout the summer or attained at lower doses. Treg population, defined as CD25^hi^CD127^−^CD4^+^ T cells, did not change their numbers, although higher FoxP3 expression was observed in the summer when solar UVB and vitamin D levels are at their peak (19). Seasonal cycles have been described in other immune markers (20) and may help explain the seasonal incidence of illnesses e.g. wintertime influenza (21), and mortality in a natural population (22), as well as the seasonal incidences of some autoimmune conditions (e.g. inflammatory bowel disease) (23), but the contribution of vitamin D is uncertain.

The primary aim of this study was to investigate the effect of oral vitamin D_3_ at UK population-recommended doses on nTreg populations. We investigated whether treating healthy adults with 400 IU vitamin D daily would increase nTregs within 12 weeks and whether any increase would be maintained for 10 months, independently of seasonal changes. We also assessed T cell proliferation and PBMC’s-derived IL-10 and IFN-γ production in response to control stimuli.

## Methods

### Study Population

Healthy adults (>18 years) from the Aberdeen area in NE Scotland (57°N) were recruited to this double-blind, randomized, placebo-controlled trial using local advertising and screened by telephone or personal interview. People taking ≤ 200 IU/day vitamin D at screening were eligible if they discontinued the supplements at least 1 month before baseline.

Exclusion criteria included being pregnant or lactating, receiving immunosuppressive or regular use of anti-inflammatory medication, or continuing to take supplements containing >100 IU vitamin D. Anyone with an immune-mediated disorder, rheumatoid arthritis, multiple sclerosis, asthma, hay fever, lupus or eczema; kidney disease; cancer or other serious immune compromising conditions, or who planned holidays abroad (> 1 month to sunny destinations), or had used sunbeds in the last 3 months were also excluded.

Full written informed consent was obtained from all participants and ethical approval for this study was granted by the College of Life Sciences and Medicine Ethics Review Board (CERB), University of Aberdeen. The procedures followed were under the Helsinki Declaration of 1975 as revised in 1983. The study was registered at the ISRCTN registry for clinical research trials (ISRCTN: 73114576).

### Intervention

Participants were randomly assigned to receive one capsule of 400 IU vitamin D_3_ (UK RNI dose, 10μg cholecalciferol; n = 31 VitD_3_ group) or placebo (n = 31 placebo group) daily for 43 weeks. Permuted block randomization (size of 8) was conducted using a web-based system managed by the Health Service Research Unit, University of Aberdeen. The Vitamin D_3_ was provided by Pure Encapsulations (Sudbury, MA) and matching placebo by Cultech, UK. Capsules were opaque, contained the same excipient (blend of fructooligosaccharides, microcrystalline cellulose, silica, and magnesium stearate) and were similar in size and color. Compliance was assessed by counting leftover capsules at the end of the intervention period. Participants and investigators were blinded to the intervention’s treatments.

Participants attended the Health Sciences Building at the University of Aberdeen for assessment at 5-time points: starting in March 2015 with baseline (March, spring), 4 weeks (April, spring), 12 weeks (June, summer), 25 weeks (September, autumn) in 2015 and the last visit at 43 weeks in January 2016 (winter). Baseline assessment included demographic questions, age, height, weight, skin color using CM-2600d Spectrophotometer, dietary vitamin D intake and a sunlight questionnaire to estimate seasonal sunlight behaviors (time spent outside and body surface exposure for each season).

### Venesection and Blood Processing

Subjects provided 45mL fasted blood samples at each visit collected into the following tubes: Lithium heparin sulfate tubes (3 x 9mL) and serum activated clot VACUETTE^®^ tubes (1 x 9mL) (Greiner Bio-One, Austria) for the analysis of immune markers, Serum Separator Tubes (1 x 5mL, SST™ tube) and EDTA tubes (1 x 4mL) both from BD Vacutainer^®^ (UK) for vitamin D metabolites and PTH levels, respectively.

### Peripheral Blood Cell Preparation

Peripheral blood mononuclear cells (PBMCs) were isolated from heparinized venous whole blood by density-gradient centrifugation (Lymphoprep, Axis-Shield, Oslo, Norway). PBMCs were resuspended in Alpha modification of Eagle’s medium (Gibco, Glasgow, UK), supplemented with 5% autologous serum for the measurement of immune markers.

### Tissue Culture

PBMCs were cultured in duplicates of 1mL volumes in 24-well tissue culture plates at 1.25x10^6^/mL at 37°C in a humidified atmosphere of 5% CO_2_/95% air and were stimulated for 3 days with 2.5x10^5^ beads/mL anti-CD3/anti-CD28-coated beads (Dynabeads human T-Activator, Invitrogen, Glasgow, UK) or 1% Concanavalin A (ConA, positive control) or were left unstimulated.

### Proliferation Assays

Proliferation was measured as the incorporation of ^3^H-thymidine (18.5 kBq, Amersham Biosciences, Amersham, UK) over the final 6h of a 72-h culture period. Cells were then harvested onto glass-fiber filter mats (LKB-Wallac Ltd, Turku, Finland) using a Mach III harvester and the radioactivity was measured by liquid scintillation (Microbeta+, LKB-Wallac). Proliferation results are presented as counts per minute (CPM) ± SEM of triplicate samples.

### Cytokine Production

As the cytokines were measured after the PBMC were incubated with T cell stimuli, the cellular source is likely to be predominantly T cells. IL-10 and IFN-γ concentrations in cell culture medium were analyzed by enzyme-linked immunosorbent assay. All antibodies and reagents for this assay and nTreg quantification were supplied by Becton Dickinson Pharmingen (San Diego, California) unless otherwise stated. The antibody pairs for human cytokines were anti‐IFN‐γ (clones NIB42 and 4S.B3 BD) and anti‐IL‐10 (clones JES3-19F1 and JES3-12G8), used at 2 μg/L and 1 μg/mL, respectively. Cytokine concentrations were measured in 100µL duplicate samples of 3-day stimulated PBMC culture supernatants. Reconstituted (in phosphate buffer saline containing 5 mg/mL bovine serum albumin) recombinant human IFN-γ (500 μg/mL) and Recombinant Human IL-10 (100 μg/mL) were used for generating standard curves. The wells were developed with 1/10,000 ExtraAvidin^®^-alkaline phosphatase conjugate and phosphatase substrate (Sigma-Aldrich, USA), and absorbance determined at 405 nm using a Multiskan™ plate reader (Labsystems, Basingstoke, UK). The standard curves were modelled by a smoothed cubic spline function, which was applied to the absorbance readings and the cytokine concentrations after a quasi-logarithmic transformation, where: quasilog_e_ = log_e_ [z + √ [z^2^ + 1]].

### Natural Treg Quantification

Freshly isolated PBMCs were stained using the following fluorescentally-labelled monoclonal antibodies: anti-CD3-FITC (clone HIT3a, 20μl/test), anti-CD4-PerCP-CY5.5 (clone RPA-T4, 5μl/test), anti-CD25-APC (clone M-A251, 5μl/test), anti-CD127-PE-CY7 (clone HIL-7R-M21, 5μl/test). Cells were also fixed and permeabilized as per manufacturer’s protocol using the human FoxP3 buffer Set before the intracellular staining of Human FoxP3 by anti-FoxP3-PE (clone 259D/C7, 20μl/test). All data were acquired using the LSR II (BD Biosciences, San Diego, California) and analyzed using FlowJo v.9.3.1 (Tree Star, Inc, Ashland, Ore). PE isotype, CD25 and FoxP3 minus one (FMO) controls were run during each experiment and compensation beads were used for each sample, providing single positive controls for voltage adjustments and compensation calculations. The Treg population investigated in this study are the thymus-derived Treg or naturally occurring Tregs (nTregs), identified as the proportion (percentage) of CD3^+^CD4^+^CD127^lo^ cells with a CD25^+^FoxP3^+^phenotype (%nTreg).

### Vitamin D Metabolites and Parathyroid Hormone

Biochemical analysis was performed at the Bioanalytical Facility (University of East Anglia, Norwich, UK). Serum 25(OH)D_3_, 25(OH)D_2_, 24,25(OH)_2_D_3_ and 24,25(OH)_2_D_2_ concentrations were determined simultaneously from a single analysis by liquid chromatography-tandem mass spectrometer as described previously (24). Briefly, serum samples extracted using supported liquid extraction plates (Biotage, Uppsala, Sweden) to de-lipidate phospholipids, followed by derivatization with 4-Phenyl-1,2,4-triazole-3,5-dione. 25(OH)D_3_ and D_2_ were calibrated using commercial standards (Chromsystems, München) traceable to standard reference material SRM972a from the National Institute of Science and Technology. The assay linearity was between 0.1-200 nmol/L. The inter/intra-assay coefficient of variation (CV) was ≤9%, the lower limit of quantification (LLoQ) of 0.1 nmol/L. The assay showed <8% accuracy bias against NIST reference method on the Vitamin D external quality assessment scheme. 24,25(OH)_2_D_3_ and 24,25(OH)_2_D_2_ were calibrated using in-house spiked standards traceable to NIST SRM972a. The assay was linear between 0.1-25 nmol/L; inter/intra-assay CV was ≤11%, LLoQ of 0.1 nmol/L for 24,25(OH)_2_D_3_ and 0.8 nmol/L for 24,25(OH)_2_D_2_. Total 25(OH)D and 24(OH)_2_D were reported as the sum of their respective (D_2_+D_3_) forms.

Serum 1,25(OH)_2_D was analyzed by chemiluminescent immunoassay using the DiaSorin LIAISON^®^ XL automated analyzer (Stillwater, MN, USA). The assay measured total 1,25(OH)_2_D between 12-480 pmol/L, the inter/intra-assay CV were ≤9.2%. On the Vitamin D external quality assessment scheme, the method showed ≤8.5% bias against method-specific mean and ≤9.1% bias against all method mean. Plasma PTH was analyzed on the COBAS platform by electrochemiluminescence immunoassay (Roche Diagnostics, Mannheim, Germany). The inter-assay CV was ≤3.8% across the analytical range of 1.2-5000 pg/mL.

### Other Analyses

Sunlight UVB exposure was measured over 7 consecutive days using UVB polysulfone film badges, one week after the baseline, weeks 4, 12 and 25 study visits. An extra measurement was taken at week 16 at the peak of the summer period in July. The absorbance of the badges pre- and post-solar UVB exposure, was measured by standard measures and the standard erythema dose was calculated as 10.7 (ΔA330) +14.3 (ΔA330)^2^−26.4 (ΔA330)^3^+89.1 (ΔA330)^4^ where ΔA330 is equal to the difference in readings (25, 26). A daily sunlight diary accompanied the badges to collect information such as the amount of time spent outdoors, and skin exposed for each day the badges were worn. Holidays taken over the study period were also recorded. Skin color (as individual topology angle) was measured at each visit on the skin surface of 4 sites in the following sequence: left and right cheekbones, and the inner and outer right forearm, as previously described (27, 28). The individual topology angle was defined using the following equation: ITA = (arctangent (L* − 50)/b*) × 180/π (29).

### Statistical Analysis

Using data that showed a mean increase in natural log %nTregs of 1.68 (SD 1.93) (27), it was estimated that 25 subjects would be required to detect the same difference in Tregs with 85% power and a significance level of 5% (PS Power version 3.0.43). Based on previous studies (3, 30), sixty volunteers (30 subjects in each arm) were recruited to allow for 20% withdrawal. Baseline characteristics were described for each group using mean and standard error mean (SEM) for normally distributed continuous variables and median (IQR) for those skewed, with number and percentage for categorical variables. A linear mixed model was used to compare the change in vitamin D metabolites with supplementation at each visit. A linear mixed model was also used to compare treatment groups (VitD_3_ versus placebo) for their immune marker response (nTregs, T cell proliferation, IL-10 or IFN-γ) over time (visit) adjusted for other confounders (age, height, weight, dietary vitamin D, sunlight exposure, a holiday abroad, and skin pigmentation). An *a priori* decision was made to conduct a sensitivity analysis including compliance >85%. Results were analyzed using SPSS version 24.

## Results

### Participants Recruitment and Baseline Characteristics

**Figure 1:**
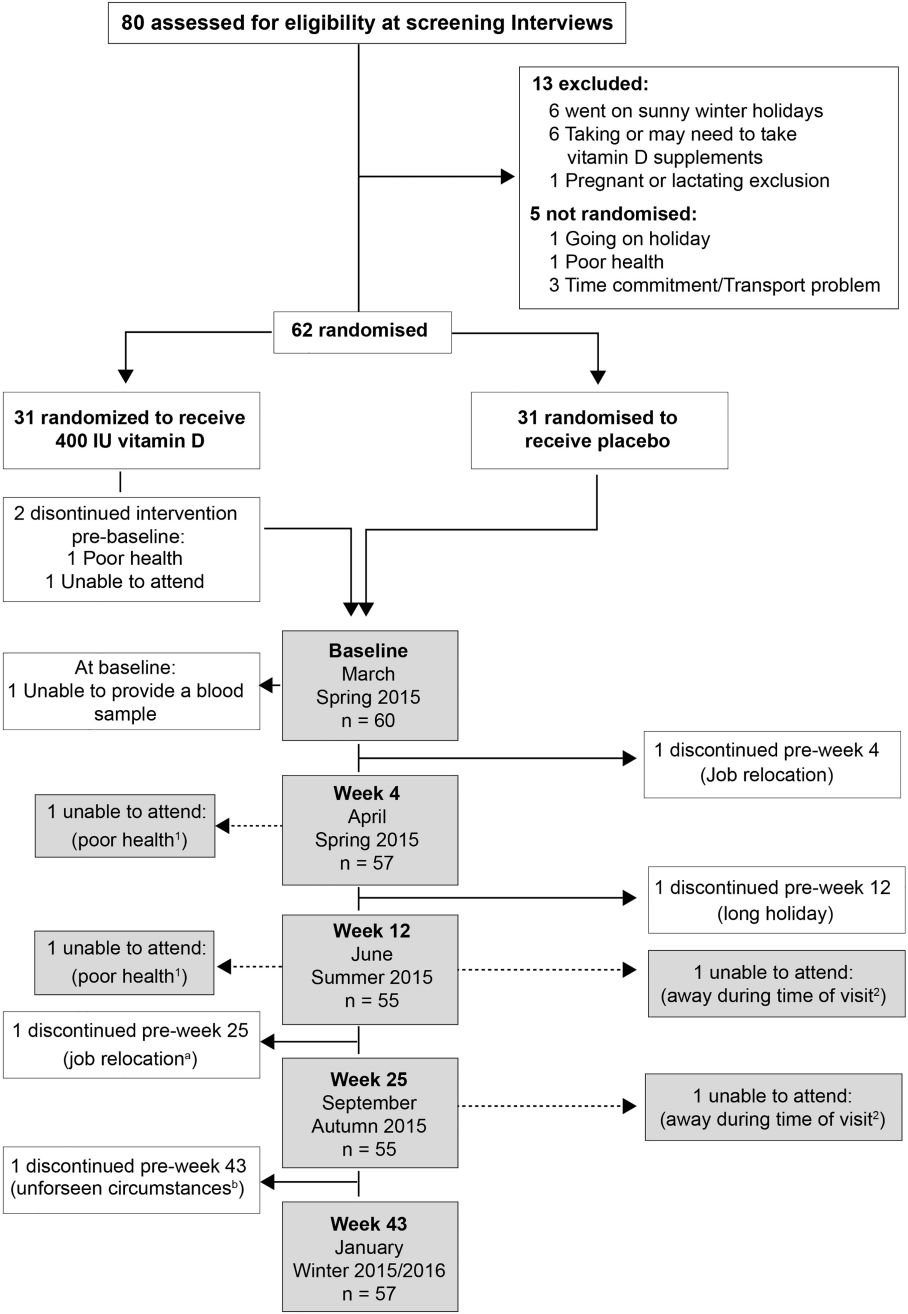
Participants’ enrolment and retention: a and b - withdrew from the follow-ups but returned for the final visit; 1 and 2 – Individuals unable to attend more than 1 study visit.

Eighty individuals were assessed for eligibility; sixty-two non-smokers met the inclusion criteria, of whom seven withdrew for the following reasons: poor health (n=1), time constraints (n=1), job relocation (n=2), blood sampling (n=1), long sunny holiday (n=1) and personal reasons (n=1) (**Figure 1**). From these seven volunteers, three others were randomized but 2 of these failed to attend the baseline visit, and the third attended but did not provide a sample and withdrew from the study. No-one reported taking supplements containing vitamin D; 9 participants (n 5, placebo vs n 4, VitD_3_) reported a winter-time holiday before study commencement; 2 participants (VitD_3_ group) reported using a sunbed (> 3 months ago). Baseline characteristics are summarized in **Table 1**. Baseline mean (SEM) serum 25(OH)D concentrations were similar between placebo (36.4(3.2) nmol/L) and VitD_3_ (35.8(3.0) nmol/L) groups. Baseline serum concentrations for 1,25(OH)_2_D, its catabolic marker 24,25(OH)_2_D and PTH were also similar between the groups. At baseline, 29% and 32% of subjects in the placebo and VitD_3_ group, respectively, had 25(OH)D concentrations < 25 nmol/L. Compliance with the intervention was similar between the groups and over 85% in both groups.

**Table 1:** Participant characteristics at baseline.

	Placebo	Vitamin D_3_(400 IU/d)	All
**n**	**31**	**28**	**59**
Females, n (%)	19 (61.3)	21 (75.0)	40 (67.8)
Age (y)	50.0 (3.2)	43.6 (3.2)	47.0 (2.3)
Height (cm)	168 (1.8)	165.9 (2.1)	167.0 (1.3)
Weight (kg)	70.7 (2.2)	67.7 (2.6)	69.3 (1.7)
BMI (kg/m2)	25.0 (0.7)	24.6 (0.9)	24.8 (0.5)
Dietary vitamin D (µg/day)	2.6 (0.3)	3.3 (0.4)	3.0 (0.2)
Holiday abroad previous winter, n (%)	5 (16.1)	4 (14.3)	9 (15.3)
Cheek skin color (ITA)	41.8 (1.5)	43.2 (1.7)	42.4 (1.1)
Right Inner arm skin color (ITA)	54.4 (7.2)	51.9 (8.8)	53.5 (8.7)
Right outer arm skin color (ITA)	48.6 (1.1)	48.7 (1.8)	48.6 (1.0)
25(OH)D (nmol/L)	36.4 (3.2)	35.8 (3.0)	36.1 (2.2)
PTH (pmol/L)	5.3 (0.3)	4.8 (0.2)	5.1 (0.2)

Number (%) for categorical variables (SEM). Mean (SEM) for continuous variables.

ITA, individual topology angle.

### Sun Exposure and Skin Pigmentation

Median (IQR) of the standard erythema dose in the placebo group increased from 1.7(3.6) in March, to 3.7(6.7) in April (4w), 4.9(5.8) in June (12w), 3.7(5.0) in July (16w), and decreased to 2.2(2.1) in September. Similarly, weekly UVB exposure in March was low in the VitD_3_ group, peaked in the summer before declining in the autumn. There was no significant difference in sunlight UVB exposure between the groups (data not shown).

Skin pigmentation was also not statistically different between groups and varied similarly over time in both groups as darker skin pigmentation was measured in April, June and September compared to baseline (p<0.001, at each timepoint). For the placebo group, the mean (SEM) skin pigmentation in March decreased over the summer from an ITA of 41.8(1.5), reaching the nadir (greatest skin darkening) in September (36.5(1.6)), before returning to baseline the following January (40.7(1.0)). Skin pigmentation for VitD3 in September was 38.3(2.0), 13% lower than baseline value in March (43.2(1.7)).

### Vitamin D Metabolites and PTH

**Figure 2:**
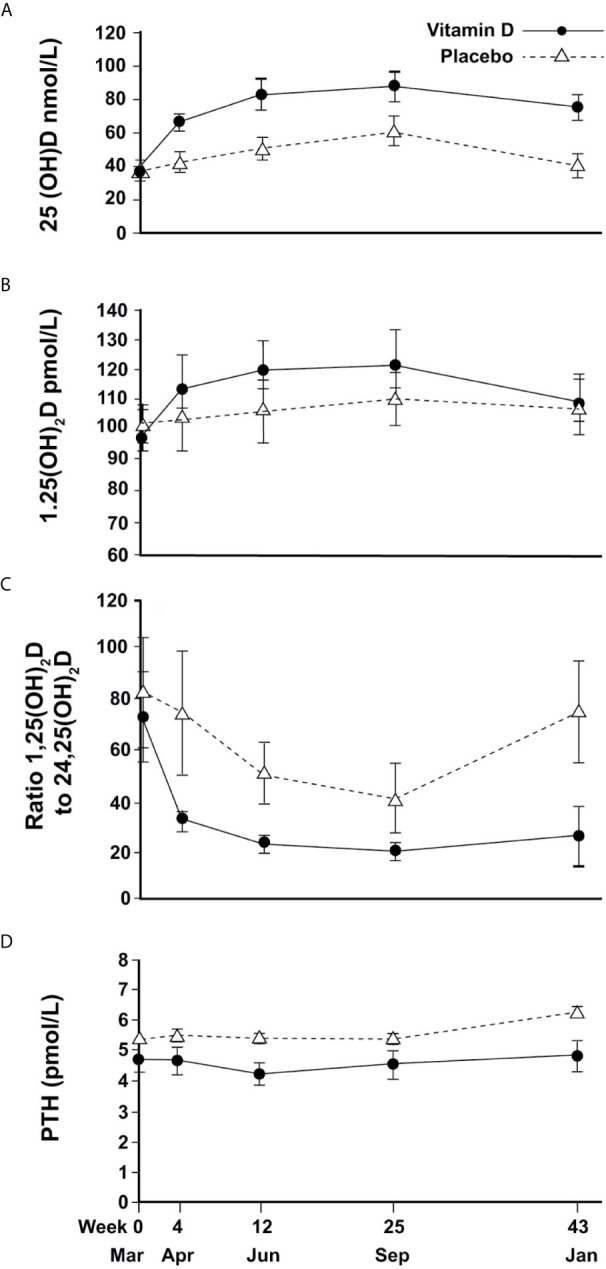
Changes in circulating vitamin D metabolites and PTH (mean and 95%CI error bars) over time; **(A)** 25(OH)D, **(B)** 1,25(OH)^2^D, **(C)** Ratio of 1,25(OH)^2^D to 24,25(OH)^2^D, **(D)** PTH.

In the placebo group, mean (SEM) 25(OH)D concentrations changed seasonally, increasing to 40(3.0) nmol/L in April (4w), 50(3.5)nmol/L in June (12w), 60(4.6) nmol/L in September (25w) and returning to baseline (40(3.5) nmol/L) in January (43w). Vitamin D treatment resulted in an 82% increase in 25(OH)D to a mean of 65(3.0) nmol/L in April (4w) with a plateau of around 80-85 nmol/L in June and September before decreasing slightly to 75(4.0) nmol/L the following January (**Figure 2A**). Vitamin D supplementation showed a similar pattern of increased 24,25(OH)_2_D concentrations compared to placebo (data not shown). For both metabolites, mixed models showed significant time-treatment interactions (P<0.001, at each time point from April to January).

For 1,25(OH)_2_D, a change over time was observed but with no significant difference between treatment and placebo (**Figure 2B**).

The ratio of 1,25(OH)_2_D to 24,25(OH)_2_D varied seasonally for the placebo group, reaching a trough of 40 in September and returning to baseline in January, whereas the VitD_3_ group had a consistently lower value that plateaued at a ratio of 20 from June until the following January. The time-treatment interactions were significant at each time point (April P<0.001, June P=0.001, September P=0.02, January P<0.001) (**Figure 2C**). Plasma PTH concentration did not change with time although treatment resulted in a marginal reduction overall (p=0.02) (**Figure 2D**).

### nTregs and T Cell Proliferation

**Figure 3:**
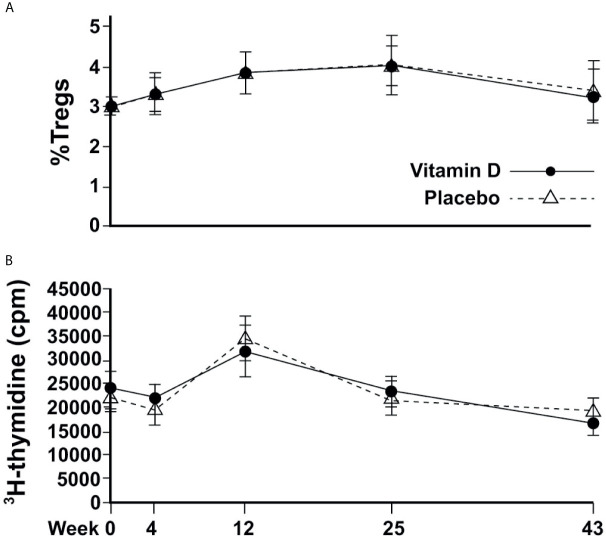
Changes in immune cell responses over time. **(A)** Circulating proportion of Tregs; **(B)** T-effector cell proliferation.

The %nTregs and T cell proliferation values were similar between treatment groups at baseline. There was no significant association between serum 25(OH)D concentrations (or the concentrations of 24,25(OH)_2_D, 1,25(OH)_2_D, or ratios of vitamin D metabolites) and baseline %nTregs. Vitamin D supplementation did not appear to affect nTregs, although there was a seasonal increase in summer that was very similar to the placebo (**Figure 3A**). The %nTregs (placebo group) increased gradually from a mean (SEM) of 2.9(0.2)% in March to peak in June and September to 3.8(0.3)% and 4.0(0.4)%, respectively, then decreasing to 3.4(0.4)% in January. The seasonal pattern remained significant (P<0.001 for June, September and January) after adjustment for confounders, with skin color (P<0.001) and body weight (P=0.015) being additional predictors.

There was no correlation between 25(OH)D or any other vitamin D metabolite and induced T cell proliferation at baseline; and vitamin D supplementation did not appear to affect proliferative responses. T cell proliferation was similar between groups at every time point, and followed similar changes according to the season, from a mean of 21851(1398) cpm and 19380(1536) cpm in March and April, respectively, to a significantly higher response in June of 34463(2359), before returning to 21880(1794) and 19207(1458) in September and January, respectively for placebo (**Figure 3B**). Mixed modelling showed significant differences from baseline in June (P<0.001) and January (P<0.001) after adjustment for confounders, with PTH being an additional predictor (P=0.027).

### Stimulated-Cell Cytokine Production

**Figure 4:**
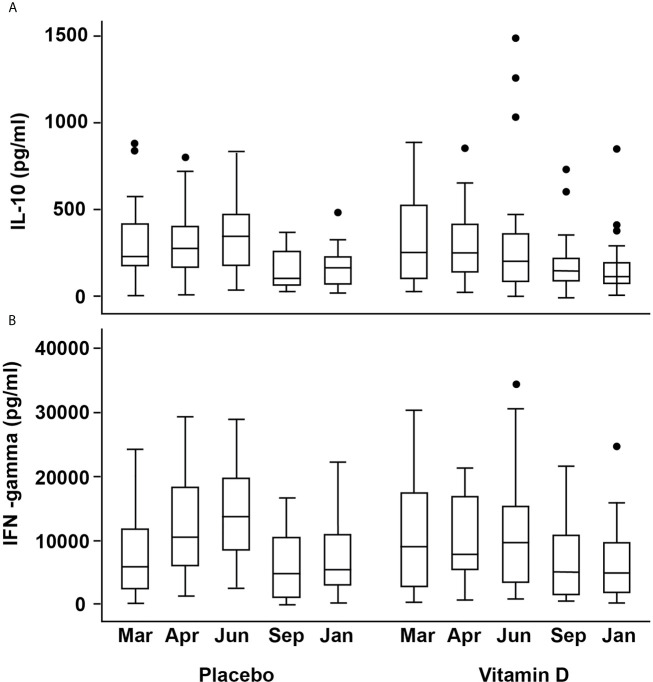
Box plots showing changes in activated cell-produced cytokine concentrations over time **(A)** IL-10 **(B)** IFN-γ. The box plots follow the conventional format with the bottom and top of the box corresponding to the 25th and 75th centile, respectively; the internal bar being the median; the whiskers, 1.5X the box height, with the outlying dots denoting the outliers i.e. cases that do not fall within the whiskers.

There was a weak positive association between 25(OH)D (but not other vitamin D metabolites) and secreted IL-10 concentrations from stimulated PBMCs at baseline (Spearman’s r 0.270, p=0.044). There was no significant effect from vitamin D supplementation, but seasonal differences were broadly similar between the treatment groups (**Figure 4A**). In the placebo group, median (IQR) IL-10 concentrations for March, April, June were 228(248), 271(230), 344(304) pg/ml, respectively, which were higher than those for September and January (101(207) and 165(161), respectively. The vitamin D_3_ group showed median (IQR) for IL-10 for March, April, June of 250(433), 248(283), 202(278) in pg/ml compared to 143(148) and 115(120) in pg/ml in September and January, respectively. Mixed modelling showed IL-10 concentrations to be significantly greater in June compared to baseline (P<0.001), after adjustment for confounders and that dietary vitamin D was an additional predictor (P=0.037, **Table 2**).

**Table 2:** Mixed Model Analysis to determine predictors of IFN-γ and IL-10 produced by stimulated T-cells.

		Beta	95% CI	*p*
Outcome: IFN-γ^a^				
Intercept		80.0	64.9 to 95.1	<0.001
Independent variables				
Time (weeks) *versus* baseline				
	4 weeks	26.8	11.8 to 41.8	0.001
	12 weeks	32.6	14.6 to 50.6	0.001
	25 weeks	-12.2	-31.6 to 7.1	0.21
	43 weeks	-1.6	-16.8 to 13.5	0.829
Treatment (*versus* placebo)		12.8	-9.3 to 34.9	0.252
Interaction time * treatment				
	4 weeks * placebo	-25.6	-47.3 to -3.9	0.022
	12 weeks * placebo	-32.7	-58.8 to -6.7	0.015
	25 weeks * placebo	-5.8	-34.1 to 22.4	0.68
	43 weeks * placebo	-22.8	-44.8 to -0.9	0.042
		Beta	95% CI	*p*
Outcome: IL-10^b^				
Intercept		17.1	14.7 to 19.5	<0.001
Independent variables				
Time (weeks) *versus* baseline				
	4 weeks	-0.5	-2.9 to 1.9	0.666
	12 weeks	0.5	-2.2 to 3.2	0.699
	25 weeks	-5.0	-7.3 to -2.8	0.001
	43 weeks	-4.5	-6.6 to -2.4	0.001
Treatment (*versus* placebo)		-0.8	-4.3 to 2.7	0.650
Interaction time * treatment				
	4 weeks * placebo	0.7	-2.8 to 4.2	0.682
	12 weeks * placebo	-1.3	-5.2 to 2.6	0.508
	25 weeks * placebo	1.3	-1.9 to 4.5	0.428
	43 weeks * placebo	0.3	-2.8 to 3.4	0.838

a IFN-γ (pg/ml) square-root transformed.

Using the linear equation generated from the above, mean IFN-γ converted to ng/ml was predicted to be, at baseline, 4, 12, 25 and 43 weeks, respectively: 6.4, 11.4, 12.6, 4.6 and 6.4 for placebo compared to 8.6, 8.8, 8.6, 5.6 and 4.7, respectively for 400 IU daily vitamin D treatment. Gender and body weight were found to be additional significant predictors of IFN-γ with beta (95% CI) 16.9 (0.9 to 33.0) for female (*versus* male) and 0.7 (0.1 to 1.3) per kg body weight, with beta remaining similar for the other variables with the exception of the intercept {beta (95% CI): +20.6 (-27.6 to +68.8)}. Independent variables tested which were not significant predictors of IFN-γ included: age, 25(OH)D, PTH, dietary vitamin D, a holiday abroad and skin color.

b IL-10 (pg/ml) square-root transformed.

Using the linear equation generated from the above, mean IL10 converted to pg/ml was predicted to be, at baseline, 4, 12, 25 and 43 weeks, respectively: 292, 276, 310,146 and 159 for placebo compared to 266, 272, 240,159 and 146 for vitamin D.

The effect of vitamin D treatment was not significant. There were no additional significant predictors including gender, body weight, age, 25(OH)D, PTH, dietary vitamin D, a holiday abroad and skin color.

Similar seasonal variation was observed for IFN-γ and vitamin D appeared to attenuate the increase seen in April and June in the placebo group. The corresponding medians (IQR) in ng/ml for the placebo group were 10.5(12.2), 13.7(11.8) in April and June compared to 7.8(11.5), 9.7(11.9) for the VitD_3_ group, which equates to a reduction of 26% and 29% respectively **(** **Figure 4B** **)**. Mixed modelling showed a significant time-treatment interaction for IFN-γ (**Table 2**), which remained significant after testing for other variables. This indicates the effect of treatment differs with time and does not remain constant. Gender and body weight were found to be additional predictors of IFN-γ concentration but not age, 25(OH)D, PTH, holidays abroad, dietary vitamin D, nor skin color. Sensitivity analysis, including compliance >85%, did not affect the results of the model. Using the linear equation generated from the mixed model, mean IFN-γ concentrations (ng/ml) were predicted to be, at baseline, 4, 12, 25 and 43 weeks respectively: 6.4, 11.4, 12.6, 4.6 and 6.4 for placebo, compared to 8.6, 8.8, 8.6, 5.6 and 4.7 respectively for the 400 IU daily vitamin D treatment.

## Discussion

Our study demonstrates seasonal variation for markers of vitamin D and all immune markers tested, with summer values 13%, 46%, 50%, 56% higher than in winter for %nTregs, proliferation, IL-10 and IFN-γ, respectively. However, daily vitamin D_3_, at a physiologically relevant dose of 400IU for 43 weeks starting in March, did not affect %nTregs and other immune markers in healthy volunteers. An additional finding was an interaction between season and vitamin D treatment, whereby oral vitamin D attenuated the seasonal rise of IFN-γ observed for the placebo group in April, June, and January, but not in September (when 25(OH)D is generally at its highest). The tandem mass spec method used for the measurement of vitamin D metabolites includes a de-lipidation procedure, which results in greater recovery of 25(OH)D and allows the measurement of other vitamin D metabolites such as 24,25(OH)_2_D (included in this study) that circulate at lower concentrations (24). Using over 2000 measurements of 25(OH)D done with and without de-lipidation, the mean peak 25(OH)D values was estimated to be 13 nmol/L higher than if the de-lipidation step was not included (with less of a difference at lower values) (31). Hence, after 25 weeks treatment with 400 IU daily vitamin D, the 25(OH)D peak would be 73 nmol/L without de-lipidation. In addition, the lower starting 25(OH)D (late spring) results in a greater increase in 25(OH)D than would be seen if the starting 25(OH)D were higher.

Evidence from five clinical trials comparing oral vitamin D_3_ with a control or placebo group on %nTregs is inconsistent. Baris et al. (17) reported significantly higher %nTregs in asthmatic children receiving pharmacotherapy after 12-months of subcutaneous immunotherapy (SCIT) plus 650 IU/day vitamin D compared with those receiving SCIT alone, and Prietl et al. (16) observed 16% higher %nTregs with vitamin D (140,000 IU/month) compared to placebo in a 12-week wintertime study in healthy adults. In contrast, Gabbay *et al.* (32) observed no differences in %nTregs between groups taking 2000 IU/day vitamin D_3_ or placebo in an 18-month study of adults and children with new-onset Type 1 diabetes (T1D). Similar %nTregs were reported between treatment groups for adults with relapsing-remitting MS who underwent a 2-step intervention with daily vitamin D_3_ (7000 IU for 4 weeks, followed by 14000 IU for 44 weeks) (33). These results corroborate our findings and those of Treiber et al. (18) in children with new-onset T1D treated with ~3434 IU/day for 12 months in Austria. However, the latter study found significantly higher Treg immunosuppressive capacity in the vitamin D group, while Prietl et al. (16) found no change in Treg immunosuppressive properties despite increased %nTregs (16). Observational studies support a role for vitamin D on Treg function in some patient groups (34), and correlations between 25(OH)D and Treg suppressive functionality have been observed (18, 35), but these are subject to confounding.

Suggestions that vitamin D-mediated immunomodulatory effects are dependent on high local concentrations of 1,25(OH)_2_D have motivated the use of high doses of vitamin D (16). Our doses comply with current recommendations (2), but may not have been sufficient to drive 25(OH)D to the concentrations required to increase Treg numbers. The safety of intermittent high dose vitamin D has been questioned with an increased risk of falls and fractures (36) and lower volumetric BMD (37). Increased vitamin D resulted in concomitant increased plasma 24,25(OH)_2_D indicating increased catabolism, suggesting other feedback mechanisms to regulate 25(OH)D in addition to the regulation of circulating 1,25(OH)_2_D.

Discrepancies between studies testing oral vitamin D supplementation on %nTregs may be related to heterogeneity of study designs: the type of participants, the definition of Treg populations, dose and dosing intervals of vitamin D (from 650/day to 420,000 IU/month), and the trial duration (ranging from 3 to 18 months).

Importantly, there is a lack of information on the timing of subjects’ and controls’ recruitment to appropriately conclude on the results reported. The initial vitamin D status of the population may be important. Our study had 30% people < 25nmol/L 25(OH)D, which is typical of this Northerly latitude population in early spring, compared to 32% of people < 50nmol/L 25(OH)D for the Prietl study (16).

The physiological consequences of the attenuation of the seasonal rise of IFN-γ by oral vitamin D observed for the placebo group in April, June, and January are unclear. We used production of the cytokine IFN-γ by T cells stimulated *in vitro* as an indicator of the strength of effector responses associated with inflammation, but the ability to mount inflammation or immunity to viral infection *in vivo* will not necessarily be directly correlated with these results. The study participants were all healthy, so any conclusions about susceptibility to disease can only be suggestive without *in vivo* challenge experiments. The increase in IFN-γ observed in the placebo group might be explained by 1,25(OH)_2_D’s direct interference with the IFN-γ promoter activity (38) or its Th2-promoting action (39), which reciprocally antagonizes IFN-γ-driven Th1 responses. Mosayebi et al. (40) reported no effects of ~11,000 IU/day intramuscular vitamin D on IFN-γ after 6 months. Comparing our results with trials involving patients (41), healthy subjects (42, 43) or overweight/healthy obese subjects (44) is limited by the route of entry and dosage of vitamin D and differences in cytokine assessment, which were carried out using serum. None of the studies considered the impact of seasonal variation on IFN-γ and other cytokines. ​Seasonal variations in Treg-specific and other immune markers have been reported, but are often attributed to the seasonality of vitamin D synthesis (19, 45). We observed seasonal variations in 25(OH)D concentrations in the placebo group consistent with living at 57°N (28, 30, 46). Our study design allows us to suggest that the seasonal changes in immune markers may be independent of cutaneous vitamin D synthesis. We noted subtle differences in the seasonal variations: %nTreg followed the same pattern of 25(OH)D, which peaked in June & September; T-effector proliferation responses were highest in June, decreasing in September, whereas IL-10 and IFN-γ productions were higher in April and June than September and January. These seasonal variations confirm previous reports in non-intervention studies at 52°N in the Netherlands (19, 45), gene expression studies in UK and Netherlands cohorts (20) (47) and a randomized control trial with 2000 IU/d vitamin D in healthy participants (43).

Seasonal patterns of human immune activity may reflect responses to environmentally driven factors. Ter Horst et al. (47) reported annual seasonality of immune and cytokine production, which paralleled peaks in summer temperature and atmospheric NH_3_ and O_2_ levels, wintertime humidity, SO_2_, NO, NO_2_, and CO concentrations, and different species of pollen at different times of the year (47). Melatonin, which has immunomodulatory properties (48–50), may be key to understanding photoperiod-driven changes in host immune activity. However, evidence from seasonally controlled clinical trials is needed.

The main strengths of this study were good compliance, low dropout rates, and strict inclusion criteria to control for confounders known to affect vitamin D status and immune function. All study visits started in March, before the appearance of UVB (46). Our placebo arm was contemporaneous to the supplementation arm, and all study visits were conducted within 1 month to properly account for sunlight exposure and seasonal effects over the 10-month study. To our knowledge, no previous vitamin D supplementation study has adequately accounted for the seasonal variability in the immune system activity. The dose used is the daily dose of vitamin D recommended by the UK government for adults in the population. However, we did not directly assess Treg immunoregulatory activity, and we measured only two cytokines to explore the effects of vitamin D supplementation on ex-vivo T cell responses. It would be of interest in future studies to also enumerate other regulatory populations, such as Br1, Br3 and Breg cells, but here we chose to focus on cells with a nTreg phenotype, since these comprise the most widely studied, most numerous, and arguably most important, regulatory population in health and in many immune-mediated diseases. In addition to the Treg considered in this study, there are other regulatory populations, including T-cell subsets that arise in the periphery and secrete inhibitory cytokines, such as Tr1 (IL-10) and Th3 (TGF-β). Il-10 secreting T cells in particular have been associated with protection or recovery from allergies and autoimmune disease and may *in vivo* include both tTreg and Tr1 subsets. The production of the effector Th17 cytokine IL-17 would also have been of interest. Given the constraints of experimental design, we used IL-10 produced by PBMC incubated with T cell stimuli as an indicator of the responsiveness of such regulatory cells and IFN-γ production by stimulated T cells as an indicator of effector function, as it would have been challenging to enumerate T cells secreting inhibitory cytokines including IL-10 and TGF-β in addition to the measurements already carried out. Furthermore, other markers and signaling pathways could have been of interest in studying different types of regulatory cells and their activity such as STAT4, STAT6, T-bet and others. However, given the constraints of sample size and expense, we chose outputs that gave the clearest indications of regulation (enumeration of the classic nTreg subset in blood and production of the key inhibitory cytokine IL-10 by stimulated T cells). Lastly, the generalization of our results is limited by the lack of ethnic diversity in our study population.

In conclusion, we observed seasonality of both circulating vitamin D markers and the immune responses that we assessed. However, additional vitamin D as oral supplementation did not affect the natural seasonal variation in %nTregs, nor ex-vivo T-effector cell proliferation and IL-10 response to stimulation. The novel finding that vitamin D attenuated T cell IFN-γ production suggests that vitamin D may partly modulate the immune response in healthy adults.

## Data Availability Statement

The raw data supporting the conclusions of this article will be made available by the authors, without undue reservation.

## Ethics Statement

The studies involving human participants were reviewed and approved by the College of Life Sciences and Medicine Ethics Review Board (CERB), University of Aberdeen. The patients/participants provided their written informed consent to participate in this study.

## Author Contributions

HM designed the RCT. AO, RB and MV designed the immune function experiments. FT and HM supervised WM. WM organized and conducted the trial. WM and HW conducted laboratory analyses. WF and JT analyzed vitamin D metabolite. WM, FT, HM, SF, RB, and MV analyzed data. and WM, FT, and HM drafted the paper. All authors contributed to the article and approved the submitted version.

## Funding

Pathways to a healthy lifestyle studentship, RANK prize funding (WM), University of Aberdeen Development fund (HM), NHS endowments EA0702 (AO), Rural and Environment Science and Analytical Services Division of the Scottish government (RESAS) (FT). This research was funded in whole, or in part, by the Wellcome Trust [Grant number 094847/Z/10/Z]. For the purpose of open access, the author has applied a CC BY public copyright license to any Author Accepted Manuscript version arising from this submission.

## Conflict of Interest

The authors declare that the research was conducted in the absence of any commercial or financial relationships that could be construed as a potential conflict of interest.

## Abbreviations

FoxP3, forkhead box P3; IFN-γ, interferon-gamma; IL-10, interleukin 10; PBMCs, peripheral blood mononuclear cells; PTH, parathyroid hormone; Treg, regulatory T cell; 25(OH)D, 25-hydroxy vitamin D; 1,25(OH)_2_D, 1,25 dihydroxy vitamin D; 24,25(OH)_2_D, 24,25 dihydroxy vitamin D.

## References

[1] Wacker M Holick MF. Vitamin D - Effects on Skeletal and Extraskeletal Health and the Need for Supplementation. Nutrients (2013) 5(1):111–48. 10.3390/nu5010111 PMC3571641 23306192

[2] SACN (Scientific Advisory Committee on Nutrition). Vitamin D and Health (2016). London: TSO. Available at: https://www.gov.uk/government/groups/scientific-advisory-committee-on-nutrition (Accessed August 5, 2017).

[3] Macdonald HM Mavroeidi A Fraser WD Darling AL Black AJ Aucott L. Sunlight and Dietary Contributions to the Seasonal Vitamin D Status of Cohorts of Healthy Postmenopausal Women Living at Northerly Latitudes: A Major Cause for Concern? Osteoporos Int (2011) 22:2461–72. 10.1007/s00198-010-1467-z 21085934

[4] Dankers W Colin EM van Hamburg JP Lubberts E. Vitamin D in Autoimmunity: Molecular Mechanisms and Therapeutic Potential. Front Immunol (2017) 7:697. 10.3389/fimmu.2016.00697 28163705 PMC5247472

[5] Mutt SJ Jokelainen J Sebert S Auvinen J Järvelin M-R Keinänen-Kiukaanniemi S. Vitamin D Status and Components of Metabolic Syndrome in Older Subjects From Northern Finland (Latitude 65◦North). Nutrients (2019) 11(6):1229. 10.3390/nu11061229 PMC6628213 31151163

[6] Lang PO Aspinall R. Vitamin D Status and the Host Resistance to Infections: What it is Currently (Not) Understood. Clin Ther (2017) 39(5):930–45. 10.1016/j.clinthera.2017.04.004 28457494

[7] Morales-Tirado V Wichlan DG Leimig TE Street SEA Kasow KA Riberdy JM. 1α,25-Dihydroxyvitamin D3 (Vitamin D3) Catalyzes Suppressive Activity on Human Natural Regulatory T Cells, Uniquely Modulates Cell Cycle Progression, and Augments FOXP3. Clin Immunol (2011) 138(2):212–21. 10.1016/j.clim.2010.11.003 PMC3073680 21167785

[8] Chambers ES Suwannasaen D Mann EH Urry Z Richards DF Lertmemongkolchai G. 1α,25-Dihydroxyvitamin D3 in Combination With Transforming Growth Factor-β Increases the Frequency of Foxp3+ Regulatory T Cells Through Preferential Expansion and Usage of Interleukin-2. Immunology (2014) 143(1):52–60. 10.1111/imm.12289 24673126 PMC4137955

[9] Jeffery LE Burke F Mura M Zheng Y Qureshi OS Hewison M. 1,25-Dihydroxyvitamin D3 and IL-2 Combine to Inhibit T Cell Production of Inflammatory Cytokines and Promote Development of Regulatory T Cells Expressing CTLA-4 and Foxp3. J Immunol (2009) 183(9):5458–67. 10.4049/jimmunol.0803217 PMC2810518 19843932

[10] Farias AS Spagnol GS Bordeaux-Rego P Oliveira COF Fontana AGM de Paula RFO. Vitamin D3 Induces IDO+ Tolerogenic DCs and Enhances Treg, Reducing the Severity of EAE. CNS Neurosci Ther (2013) 19(4):269–77. 10.1111/cns.12071 PMC6493393 23521914

[11] Takahashi T Kuniyasu Y Toda M Sakaguchi N Itoh M Iwata M. Immunologic Self-Tolerance Maintained by CD25+CD4+ Naturally Anergic and Suppressive T Cells: Induction of Autoimmune Disease by Breaking Their Anergic/Suppressive State. Int Immunol (1998) 10(12):1969–80. 10.1093/intimm/10.12.1969 9885918

[12] Sakaguchi S Sakaguchi N Asano M Itoh M Toda M. Immunologic Self-Tolerance Maintained by Activated T Cells Expressing IL-2 Receptor α-Chains (CD25). Breakdown of a Single Mechanism of Self-Tolerance Causes Various Autoimmune Diseases. J Immunol (1995) 155(3):1151–64. 7636184

[13] Sakaguchi S Fukuma K Kuribayashi K Masuda T. Organ-Specific Autoimmune Diseases Induced in Mice by Elimination of T Cell Subset: I. Evidence for the Active Participation of T Cells in Natural Self-Tolerance; Deficit of a T Cell Subset as a Possible Cause of Autoimmune Disease. J Exp Med (1985) 161(1):72–87. 10.1084/jem.161.1.72 3871469 PMC2187548

[14] Barnes MS Horigan G Cashman KD Hill TR Forsythe LK Lucey AJ. Maintenance of Wintertime Vitamin D Status With Cholecalciferol Supplementation is Not Associated With Alterations in Serum Cytokine Concentrations Among Apparently Healthy Younger or Older Adults. J Nutr (2011) 141(3):476–81. 10.3945/jn.110.131516 21270359

[15] Bock G Prietl B Mader JK Höller E Wolf M Pilz S. The Effect of Vitamin D Supplementation on Peripheral Regulatory T Cells and β Cell Function in Healthy Humans: A Randomized Controlled Trial. Diabetes Metab Res Rev (2011) 27(8):942–5. 10.1002/dmrr.1276 22069289

[16] Prietl B Treiber G Mader JK Hoeller E Wolf M Pilz S. High-Dose Cholecalciferol Supplementation Significantly Increases Peripheral CD4+ Tregs in Healthy Adults Without Negatively Affecting the Frequency of Other Immune Cells. Eur J Nutr (2014) 53:751–9. 10.1007/s00394-013-0579-6 23999998

[17] Baris S Kiykim A Ozen A Tulunay A Karakoc-Aydiner E Barlan IB. Vitamin D as an Adjunct to Subcutaneous Allergen Immunotherapy in Asthmatic Children Sensitized to House Dust Mite. Allergy (2014) 69(2):246–53. 10.1111/all.12278 24180595

[18] Treiber G Prietl B Fröhlich-Reiterer E Lechner E Ribitsch A Fritsch M. Cholecalciferol Supplementation Improves Suppressive Capacity of Regulatory T-Cells in Young Patients With New-Onset Type 1 Diabetes Mellitus — A Randomized Clinical Trial. Clin Immunol (2015) 161(2):217–24. 10.1016/j.clim.2015.08.002 26277548

[19] Khoo A-L Koenen HJPM Chai LYA Sweep FCGJ Netea MG van der Ven AJAM. Seasonal Variation in Vitamin D3 Levels Is Paralleled by Changes in the Peripheral Blood Human T Cell Compartment. PloS One (2012) 7(1):e29250. 10.1371/journal.pone.0029250 22235276 PMC3250425

[20] Dopico XC Evangelou M Ferreira RC Guo H Pekalski ML Smyth DJ. Widespread Seasonal Gene Expression Reveals Annual Differences in Human Immunity and Physiology. Nat Commun (2015) 6:7000. 10.1038/ncomms8000 25965853 PMC4432600

[21] Cannell JJ Vieth R Umhau JC Holick MF Grant WB Madronich S. Epidemic Influenza and Vitamin D. Epidemiol Infect (2006) 134(6):1129–40. 10.1017/S0950268806007175 PMC2870528 16959053

[22] Stevenson TJ Prendergast BJ. Photoperiodic Time Measurement and Seasonal Immunological Plasticity. Front Neuroendocr (2015) 37:76–88. 10.1016/j.yfrne.2014.10.002.Photoperiodic PMC4405432 25456046

[23] Watad A Azrielant S Bragazzi NL Sharif K David P Katz I. Seasonality and Autoimmune Diseases: The Contribution of the Four Seasons to the Mosaic of Autoimmunity. J Autoimmun (2017) 82:13–30. 10.1016/j.jaut.2017.06.001 28624334

[24] Tang JCY Nicholls H Piec I Washbourne CJ Dutton JJ Jackson S. Reference Intervals for Serum 24,25-Dihydroxyvitamin D and the Ratio With 25-Hydroxyvitamin Established Using a Newly Developed LC-MS/MS Method. J Nutr Biochem (2017) 46:21–9. 10.1016/j.jnutbio.2017.04.005 28437713

[25] Challoner A Corless D Davis A Deane GH Diffey B Gupta S. Personnel Monitoring of Exposure to Ultraviolet Radiation. Clin Exp Dermatol (1976) 1(2):175–9. 10.1111/j.1365-2230.1976.tb01413.x 939044

[26] Diffey BL. “Human Exposure to Ultraviolet Radiation”. In: Hawk J, editor. Photodermatology, vol. p . London: Oxford University Press (1999). p. 5–24.

[27] Milliken SVI Wassall H Lewis BJ Logie J Barker RN Macdonald H. Effects of Ultraviolet Light on Human Serum 25-Hydroxyvitamin D and Systemic Immune Function. J Allergy Clin Immunol (2012) 129(6):1554–61. 10.1016/j.jaci.2012.03.001 22502796

[28] Jamil NA Gray SR Fraser WD Fielding S Macdonald HM. The Relationship Between Vitamin D Status and Muscle Strength in Young Healthy Adults From Sunny Climate Countries Currently Living in the Northeast of Scotland. Osteoporos Int (2017) 28(4):1433–43. 10.1007/s00198-016-3901-3 28083666

[29] Del Bino S Sok J Bessac E Bernerd F. Relationship Between Skin Response to Ultraviolet Exposure and Skin Color Type. Pigment Cell Res (2006) 19(6):606–14. 10.1111/j.1600-0749.2006.00338.x 17083487

[30] Wood AD Secombes KR Thies F Aucott L Black AJ Mavroeidi A. Vitamin D3 Supplementation Has No Effect on Conventional Cardiovascular Risk Factors: A Parallel-Group, Double-Blind, Placebo-Controlled RCT. J Clin Endocrinol Metab (2012) 97(10):3557–68. 10.1210/jc.2012-2126 22865902

[31] Macdonald HM Gryka A Tang JCY Aucott LS Fraser WD Wood AD. Longevity of Daily Oral Vitamin D3 Supplementation: Differences in 25OHD and 24,25(OH)2D Observed 2 Years After Cessation of a 1-Year Randomized Controlled Trial (VictORy Recall). Osteoporos Int (2017) 28(12):3361–72. 10.1007/s00198-017-4201-2 28916992

[32] Gabbay MAL Sato MN Finazzo C Duarte AJS Dib SA. Effect of Cholecalciferol as Adjunctive Therapy With Insulin on Protective Immunologic Profile and Decline of Residual β-Cell Function in New-Onset Type 1 Diabetes Mellitus. Arch Pediatr Adolesc Med (2012) 166(7):601–7. 10.1001/archpediatrics.2012.164 22751874

[33] Muris A-H Smolders J Rolf L Thewissen M Hupperts R Damoiseaux J. Immune Regulatory Effects of High Dose Vitamin D3 Supplementation in a Randomized Controlled Trial in Relapsing Remitting Multiple Sclerosis Patients Receiving Ifnβ; the SOLARIUM Study. J Neuroimmunol (2016) 300:47–56. 10.1016/j.jneuroim.2016.09.018 27806875

[34] Longhi MS Hussain MJ Mitry RR Arora SK Mieli-Vergani G Vergani D. Functional Study of CD4+CD25+ Regulatory T Cells in Health and Autoimmune Hepatitis. J Immunol (2006) 176(7):4484–91. 10.4049/jimmunol.176.7.4484 16547287

[35] Smolders J Menheere P Thewissen M Peelen E Tervaert JWC Hupperts R. Regulatory T Cell Function Correlates With Serum 25-Hydroxyvitamin D, But Not With 1,25-Dihydroxyvitamin D, Parathyroid Hormone and Calcium Levels in Patients With Relapsing Remitting Multiple Sclerosis. J Steroid Biochem Mol Biol (2010) 121(1–2):243–6. 10.1016/j.jsbmb.2010.03.001 20211254

[36] Sanders KM Stuart AL Williamson EJ Simpson JA Kotowicz MA Young D. Annual High-Dose Oral Vitamin D and Falls and Fractures in Older Women: A Randomized Controlled Trial. JAMA (2010) 303(18):1815–22. 10.1001/jama.2010.594 20460620

[37] Burt LA Billington EO Rose MS Raymond DA Hanley DA Boyd SK. Effect of High-Dose Vitamin D Supplementation on Volumetric Bone Density and Bone Strength: A Randomized Clinical Trial. JAMA (2019) 322(8):736–45. 10.1001/jama.2019.11889 PMC6714464 31454046

[38] Cippitelli M Santoni A. Vitamin D3: A Transcriptional Modulator of the Interferon-γ Gene. Eur J Immunol (1998) 28(10):3017–30. 10.1002/(SICI)1521-4141(199810)28:10<3017::AID-IMMU3017>3.0.CO;2-6 9808170

[39] Sloka S Silva C Wang J Yong VW. Predominance of Th2 Polarization by Vitamin D Through a STAT6-Dependent Mechanism. J Neuroinflamm (2011) 8:56–66. 10.1186/1742-2094-8-56 PMC3118349 21605467

[40] Mosayebi G Ghazavi A Ghasami K Jand Y Kokhaei P. Therapeutic Effect of Vitamin D3 in Multiple Sclerosis Patients. Immunol Invest (2011) 40(6):627–39. 10.3109/08820139.2011.573041 21542721

[41] Di Filippo P Scaparrotta A Rapino D Cingolani A Attanasi M Petrosino MI. Vitamin D Supplementation Modulates the Immune System and Improves Atopic Dermatitis in Children. Int Arch Allergy Immunol (2015) 166(2):91–6. 10.1159/000371350 25791938

[42] Martineau AR Wilkinson KA Newton SM Floto RA Norman AW Skolimowska K. Ifn-γ- and TNF-Independent Vitamin D-inducible Human Suppression of Mycobacteria: The Role of Cathelicidin LL-37. J Immunol (2007) 178(11):7190–8. 10.4049/jimmunol.178.11.7190 17513768

[43] Yusupov E Li-Ng M Pollack S Yeh JK Mikhail M Aloia JF. Vitamin D and Serum Cytokines in a Randomized Clinical Trial. Int J Endocrinol (2010) 2010:305054. 10.1155/2010/305054 20871847 PMC2943086

[44] Jorde R Sneve M Torjesen PA Figenschau Y Gøransson LG Omdal R. No Effect of Supplementation With Cholecalciferol on Cytokines and Markers of Inflammation in Overweight and Obese Subjects. Cytokine (2010) 50(2):175–80. 10.1016/j.cyto.2009.12.006 20122848

[45] Khoo A-L Chai LYA Koenen HJPM Sweep FCGJ Joosten I Netea MG. Regulation of Cytokine Responses by Seasonality of Vitamin D Status in Healthy Individuals. Clin Exp Immunol (2011) 164(1):72–9. 10.1111/j.1365-2249.2010.04315.x PMC3074219 21323660

[46] Macdonald HM Mavroeidi A Barr RJ Black AJ Fraser WD Reid DM. Vitamin D Status in Postmenopausal Women Living at Higher Latitudes in the UK in Relation to Bone Health, Overweight, Sunlight Exposure and Dietary Vitamin D. Bone (2008) 42(5):996–1003. 10.1016/j.bone.2008.01.011 18329355

[47] Ter Horst R Jaeger M Smeekens SP Oosting M Swertz MA Li Y. Host and Environmental Factors Influencing Individual Human Cytokine Responses. Cell (2016) 167(4):1111–24.e13. 10.1016/j.cell.2016.10.018 27814508 PMC5787854

[48] Medrano-Campillo P Sarmiento-Soto H Álvarez-Sánchez N Álvarez-Ríos AI Guerrero JM Rodríguez-Prieto I. Evaluation of the Immunomodulatory Effect of Melatonin on the T-cell Response in Peripheral Blood From Systemic Lupus Erythematosus Patients. J Pineal Res (2015) 58(2):219–26. 10.1111/jpi.12208 25612066

[49] Álvarez-Sánchez N Cruz-Chamorro I Díaz-Sánchez M Sarmiento-Soto H Medrano-Campillo P Martínez-López A. Melatonin Reduces Inflammatory Response in Peripheral T Helper Lymphocytes From Relapsing-Remitting Multiple Sclerosis Patients. J Pineal Res (2017) 63(4):e12442. 10.1111/jpi.12442 28793364

[50] Farez MF Mascanfroni ID Méndez-Huergo SP Yeste A Murugaiyan G Garo LP. Melatonin Contributes to the Seasonality of Multiple Sclerosis Relapses. Cell (2015) 162(6):1338–52. 10.1016/j.cell.2015.08.025 PMC4570563 26359987

